# Simultaneous Removal of Pollutants and Recovery of Nutrients from High-Strength Swine Wastewater Using a Novel Integrated Treatment Process

**DOI:** 10.3390/ani10050835

**Published:** 2020-05-12

**Authors:** Soomin Shim, Arif Reza, Seungsoo Kim, Naveed Ahmed, Seunggun Won, Changsix Ra

**Affiliations:** 1 Department of Animal Industry Convergence, College of Animal Life Sciences, Kangwon National University, Chuncheon 24341, Korea; smshim@kangwon.ac.kr (S.S.); reza.arif@kangwon.ac.kr (A.R.); seung_su89@nate.com (S.K.); naveed.uspcasw@faculty.muet.edu.pk (N.A.); 2 Department of Environmental Science, College of Agricultural Sciences, IUBAT—International University of Business Agriculture and Technology, Dhaka 1230, Bangladesh; 3 U.S. Pakistan Center for Advanced Studies in Water, Mehran University of Engineering and Technology, Jamshoro 76062, Sindh, Pakistan; 4 Department of Animal Resources, College of Life and Environmental Science, Daegu University, Gyeongsan 38453, Korea; swon@daegu.ac.kr

**Keywords:** novel treatment sequence, swine wastewater, pollutants removal, nutrient recovery, struvite

## Abstract

**Simple Summary:**

Due to the increasing trend of swine consumption in recent decades, swine husbandry practices have become more intensive in Korea. Intensive swine farming practices inevitably result in an increment of wastewater production. Treatment of high strength swine wastewater (SWW) is therefore becoming a matter of concern in Korea. Moreover, with the increasing number of swine heads, swine farms are having issues with malodor, sanitation, and disease control. In this study, a novel integrated treatment process was tested for the simultaneous removal of pollutants and nutrient recovery from high strength swine wastewater. The integrated treatment process used in this study successfully removed the nutrients and other pollutants through biological treatment, recovered the nutrients using struvite crystallization process and decolorized as well as disinfected the effluent before discharge into water bodies by electrochemical treatment. Therefore, using the proposed integrated treatment process, it might be possible to ensure efficient SWW management along with societal and environmental sustainability.

**Abstract:**

In this study, a novel treatment approach combining biological treatment, struvite crystallization, and electrochemical treatment was developed and its efficiency for the simultaneous removal of pollutants and recovery of nutrients from high strength swine wastewater (SWW) was verified. For all the parameters, maximum removal efficiencies in the lab-scale test were obtained in the range of 93.0–98.7% except for total solids (TS) (79.4%). Farm-scale process showed overall removal efficiencies for total nitrogen (TN), total phosphorus (TP), soluble total organic carbon (sTOC), and color as 94.5%, 67.0%, 96.1%, and 98.9%, respectively, while TS, suspended solids (SS), ammonium nitrogen (NH_4_-N), and ortho-phosphate (O-P) concentrations were reduced by 91.5%, 99.6%, 98.6%, and 91.9%, respectively. Moreover, the struvite recovered from SWW showed heavy metal concentrations within the range of the Korean standard for fertilizers and feedstocks and thus, suggesting its potential application as fertilizer and in animal feed production. Using the proposed process, the SWW was converted to liquid compost as a quick-acting fertilizer, struvite as a slow-release fertilizer, and the decolorized and disinfected effluent after electrochemical treatment was safe for discharge according to Korean standard. Therefore, the novel integrated treatment process used in this study can be considered as a solution for SWW management and for the simultaneous removal and recycling of nutrients (N and P).

## 1. Introduction

The swine production worldwide has excessively increased with the growing as the demand for meat and meat products [1]. According to the Statista, there were around 781 million swine heads in the world in 2018 making it the fastest growing livestock industry and, of the total swine population, more than 80% were concentrated in European Union (EU), East Asia, and America [2]. Swine production in South Korea (11.2 million) compared to the population (51.5 million) is very high and generates highly polluted swine wastewater (SWW). SWW treatment is therefore a high priority issue in Korea.

In general, the common treatment method of nutrient-rich SWW is land spreading as bio-fertilizer. However, fertilizer application above requirement and land spreading of SWW often cause nutrient accumulation in the soils. Although some of the nutrients are utilized by the plants for growth and development, most of the nutrients are either lost by leaching and runoff into groundwater and surface water, respectively and cause water pollution [3,4]. Therefore, to prevent nutrient accumulation in soil and following water pollution by SWW and chemical fertilizer usage, (1) the nutrients in SWW should completely be removed before discharge into the environment and (2) the amount of fertilizer application in soil should be reduced.

Biological treatment of SWW requires conversion of ammonium nitrogen (NH_4_-N) and nitrogen oxides (NO_x_) to nitrogen gas (N_2_) along with the removal of organic carbon (C) at high or low dissolved oxygen (DO) levels. Biological treatment methods include aerobic processes (activated sludge [3], biofilters [5], oxidation ditches [6]), anaerobic digestion [7], or anoxic/oxic processes [8], but usually have lower nutrient removal efficiencies (i.e., total nitrogen (TN) 20–60% and total phosphorus (TP) 0–50%) [9]. Deng et al. found that a post-treatment of digested SWW using sequencing batch reactor can remove about 10% of chemical oxygen demand (COD), 50% of NH_4_-N, and showed scarce removal of TP [9]. Simple continuous aeration at low redox potentials removed 32.3% of NH_4_-N and 75% of ortho-phosphate (PO_4_-P), whereas the removal efficiency reduced to half while using the intermittent aeration [10].

Phosphorus (P) is an essential element for all living organisms. All forms of P used are mined from phosphate rock that is a finite resource. P reserves can be exhausted within the next 90–300 years, with an annual increase in demand of 2% [4,11]. P recovery from livestock wastewater could be a sustainable alternative to commercial P sources as well as protect the environment. In case of nutrient removal from SWW, engineered processes of nutrient recovery would ultimately be more useful than simple land spreading for countries like Korea with limited arable lands in terms of nutrient management as well as reduce the use of chemical fertilizer [5]. Struvite crystallization is one of the most widely used and effective ways to recover nutrients from wastewater containing high concentration of nutrients [12]. Several researchers have recovered struvite from various waste streams and reported that it is equally effective as commercial phosphate fertilizer as well as feed additives [13,14,15,16]. Struvite is a crystal of equimolar concentrations of magnesium, ammonium, and phosphate and is formed in the prevailing alkaline conditions per Equation (1) [17].

Mg^2+^ + NH_4_^+^ + H_2_PO_4_^−^ + 6H_2_O → MgNH_4_PO_4_·6H_2_O + 2H^+^, (1)

Swine wastewater generally contains a higher amount of NH_4_-N than PO_4_-P and ultimately results in low NH_4_-N removal during struvite crystallization [18]. The additional input of P and Mg are therefore needed to increase the NH_4_-N removal efficiency, which involves an increase in operational cost [19]. Recently, electrochemical oxidation of wastewaters containing recalcitrant pollutants has caught the interest of researchers. It has successfully applied in treating domestic sewage [20], textile effluent [21], and tannery waste [22] and reported as simple, reliable, cost-effective and promising technique. When the electrochemical process is used as final polishing method as some of the pollutants (ammonium [23], sulfide [24], and organics [25]) can be oxidized through direct or indirect oxidation [26] and therefore has the potential to be used for treating the effluents of struvite crystallization process before discharge into the environment. Several oxidizing agents such as Fenton’s reagent, peroxide, sodium chloride, chlorine etc. are known to enhance the electrolytic oxidation. Chlorine (Cl) evolution is actively generated using an oxide electrode, and therefore both are generally used together. At the anode, Cl gives rise to chlorine gas (Cl_2_) which combines with water to form very potent oxidizing agent hypochlorous acid (HOCl) and hypochlorite ion (OCl^−^) [26].

2Cl^−^ → Cl_2_ + 2e^−^, (2)

Cl_2_ + H_2_O → HOCl + H^+^ + Cl^−^, (3)

HOCl → H^+^ + OCl^−^, (4)

Generally, swine farms in Korea are willing to construct a simple biological treatment process for ease of maintenance. According to the Korean Statistical Information Service (KOSIS), more than half (approximately 52.5%) of the swine farms in Korea have on-farm SWW treatment facilities. Among those, around 93.2% of the farms are using aerobic biological treatment process for treating SWW [27]. Therefore, despite an energy-efficient unitary process, anaerobic digestion is not considered a viable option. Farmers prefer to store the biologically oxidized SWW in the storage tanks and later apply in the arable lands as fertilizer to improve the soil properties in spring and autumn. While in summer and winter, when the top dressing is not possible, the oxidized SWW is discharged to water bodies after additional treatments. Moreover, during solid–liquid separation, the swine farms try to avoid using inorganic coagulants (like ferrous sulfate, alum etc.) due to toxic sludge production and ineffectiveness in removing heavy metals, as the Korean government has some strict regulations against using heavy metals containing liquid fertilizer in arable lands. The development of an on-farm integrated treatment process considering the situation of the farms is therefore necessary to protect the environment and ensure sustainability. In this study, a novel integrated treatment process composed of biological treatment, struvite crystallization, and electrochemical treatment was developed and tested in the lab and farm-scale to suggest the suitable and effective SWW management process in the Korean swine farms. The process utilizes biological treatment for liquid compost production, struvite crystallization for nutrient recovery and electrochemical oxidation as a final polishing treatment before discharge into water bodies.

## 2. Materials and Methods 

### 2.1. Collection of Swine Wastewater

The SWW used for the lab-scale experiment in this study was collected from a piggery (longitude 127°42′23.9″ E and latitude 37°49′40.1″ N) situated in Chuncheon, South Korea and stored at 4 °C until use. The characteristics of the collected SWW are shown in Table 1A. The farm-scale process utilized the SWW generated at the Kangwon National University swine farm (longitude 127°46′56.9″ E and latitude 37°56′23.7″ N) and its characteristics are listed in Table 1B. 

### 2.2. Configuration of the Treatment Sequence and Operational Methods

#### 2.2.1. Lab-Scale Treatment Process

**Figure 1:**
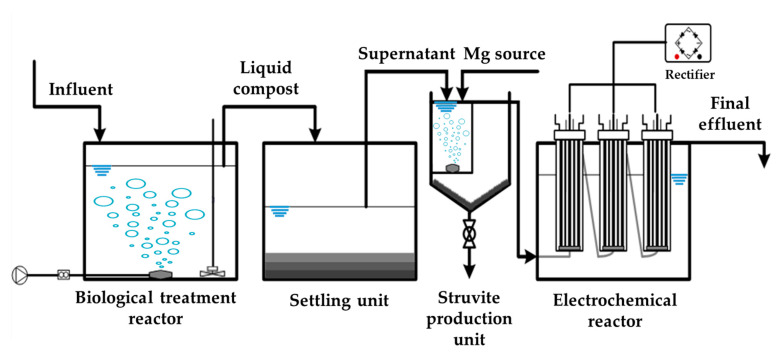
Schematic of the lab-scale treatment process.

**Figure 2:**
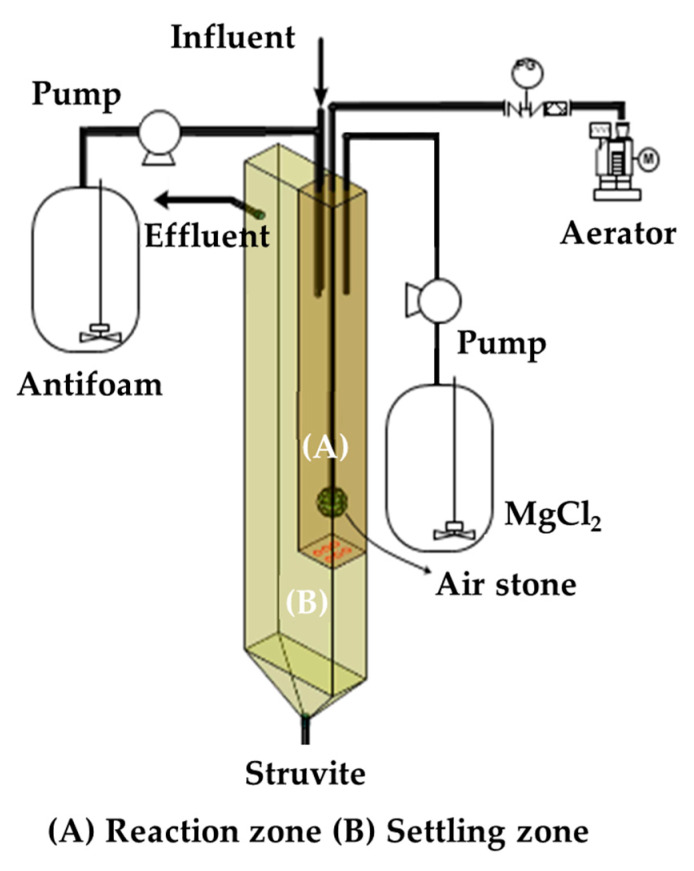
Schematic of the struvite production unit.

The sequential treatment of wastewater was composed of three steps (1) biological treatment (2) struvite production, and (3) electrochemical treatment. The schematic diagram of the treatment process is shown in Figure 1. The capacity of the reactor for biological treatment was 400 L with an effective volume of 300 L. The hydraulic residence time (HRT) was 20 d, and the whole process was operated with continuous flow mode. Intermittent aeration (2 m^3^/m^3^.min) and agitation were done every 3 h. Effluent from the biological reactor was kept in a settling unit prior to nutrient recovery. The struvite production unit (height: 50 cm, length: 10 cm, width: 10 cm) as depicted in Figure 2 was made up of Plexiglas reactor. Air was supplied using an aerator and released through an air stone diffuser. The working volume of the reactor was 3.8 L and comprised of two zones, (A) reaction zone (approximately 2.7 L) and (B) settling zone. Influent, Mg, and antifoam (Foamend CA-110, Cheong San Chem Tech Co. Ltd, Gunpo, South Korea) were continuously pumped into reaction zone, so all contents were mixed in the reaction zone and reacted in the presence of continuous air supply to form struvite crystals. HRT of the struvite recovery reactor was 6 h, and the aeration rate was fixed at 0.06 m^3^/m^3^. The reaction zone had small pores (5 mm) at the bottom to ensure the flow of liquid, solids, and crystals into the settling zone. A spigot was installed at the bottom of the reactor for struvite collection. Five spigots were installed to maintain reactor volume and effluent decanting. MgCl_2_ was added with a molar ratio of 1.2 depending on the concentration of soluble phosphate. Three electrochemical reactors were prepared using Plexiglas with a cylindrical shape (height: 120 cm, distance: 7.4 cm) and spigots were installed at top (effluent) and bottom (influent) of the reactor. The effective volume of the electrochemical reactor was maintained according to the working time for each treatment and was set at 10, 12, 14, and 16 h for experiments. Mesh type anode was titanium (Ti) plate coated with iridium (IV) oxide (IrO_2_) (width: 1 mm) and area was fixed at 93 cm^2^/L. The cathode was made up of two stainless steel plates (width: 1.2 mm), arranged side by side having the anode at the middle (distance: 1 cm). Acrylic and silicone gel were used to seal apex of the reactor. A DC power supply unit (DC 12 V 30 A, Model: WER 312, LS Electric, Anyang, South Korea) was connected with the electrodes to provide electricity. Voltage was set at 7 V, and electrolyte concentrations were optimized at 0.06% sodium chloride (NaCl) based on previous studies [28].

#### 2.2.2. Farm-Scale Treatment Process

**Figure 3:**
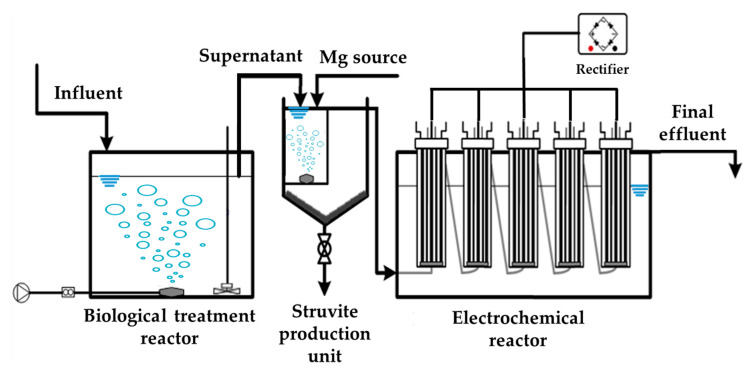
Schematic of the farm-scale treatment process.

A similar upscale treatment process was constructed at the Kangwon National University swine farm for further research and development (Figure 3). The aeration unit and the agitator were installed in the SWW storage tank with a total capacity of 100 tons to equip the developed process and converted into a sequencing batch reactor (SBR). The working volume of the SBR was 70 m^3^ with the HRT of 35 d (2 m^3^/d). The SBR was operated with the sequence of inflow (0.5 h) → anoxic stirring (4 h) → aeration (7 h) → anoxic stirring (4 h) → aeration (7 h) → settling (1 h) → discharge (0.5 h) into a tank (5 m^3^). The liquor in tank was pumped into the struvite production unit (working volume 400 L; HRT 6 h) continuously at a rate of 83.3 L/h (2 m^3^/d), and then the effluent was passed through the electrochemical reactors. The electrochemical reactor was constructed by connecting five electrolytic cells of 166.7 L cylindrical structure, and total capacity was 833.3 L. The flow of water into each electrolytic cell was done in the form of up-flow. HRT of each reactor was 2 h (total HRT 10 h) considered by results of lab scale test, and the electrode area was 93 m^2^/L. The electrolyte concentration (NaCl) was 0.06%, and the voltage was 7 V. 

### 2.3. Sampling and Analyses

Samples from influents and effluents were collected for each treatment unit and then preserved and refrigerated prior to analysis. Analyzed parameters were TS, SS, NH_4_-N, O-P, TN, TP, sTOC, color, and pH. NH_4_-N, O-P, TN, and TP were analyzed using an auto-analyzer (Quik Chem 8000, Lachat, Milwaukee, Wisconsin, USA). sTOC was analyzed using a TOC analyzer (TOC-500, Shimadzu, Kyoto, Japan) after filtration and color was analyzed by a spectrophotometer (Optizen 2120, Mecasys Co. Ltd., Daejeon, South Korea) at 400 nm. The struvite was identified and examined using SEM (S3500, Hitachi, Tokyo, Japan) and XRD (PANalytical X’Pert PRO MPD, Malvern Panalytical BV, Almelo, The Netherlands). The spread plate method was used for microbiological analysis. All analyses were done following standard methods [29].

## 3. Results and Discussion

### 3.1. Performance of the Lab-Scale Treatment Process

The pollutant concentrations of the SWW treated in this study were very high. The overall performance of the lab-scale process from SWW (influent) → biological treatment → struvite production → electrochemical treatment (16 h) is shown in Table 2 (A-G). The whole process showed 79.4% and 98.8% removal efficiencies for TS and SS, respectively. The NH_4_-N and TN removal were 98.7% and 93.9%, respectively, while the overall O-P and TP removal efficiencies were 94.7% and 93.0%, respectively. Around 94% sTOC and 97.7% color were removed in this sequence. Unit specific performances of the process are discussed below. 

#### 3.1.1. Biological Treatment Unit

Aerobic treatment is the most widely used biological treatment method in Korea. The organic material decomposition rate under the aerobic process is much faster than anaerobic process. Moreover, the application of liquid compost produced by anaerobic treatment is limited in Korea due to biological safety issues such as possible pathogenic contamination and crop productivity inhibition. In addition, after anaerobic treatment, further aerobic treatment is required for wastewater with high organic material. Therefore, considering the above context, the aerobic biological treatment process was used in this study. 

In this study, the initial pH of SWW was slightly alkaline (~7.5), and it was increased over 8.6 through air stripping. The pH was increased due to the buffering capacity of the SWW. The solids removal efficiency of the biological and settling unit was 52.7% and 77.6% for TS and SS, respectively. The initial NH_4_-N concentration was decreased from 3454 mg/L to 989 mg/L. Most of the NH_4_-N was removed during the intermittent aeration with the removal efficiency of 71.4% (Table 2). While the TN concentration was decreased from 11,362 mg/L to 7891 mg/L and showed lower (30.5%) removal efficiency than NH_4_-N. The NH_4_-N concentration in the SWW was comprised of 30.4% of TN. Assuming all of the NH_4_-N was removed (30.5% TN) in the biological reactor, there was still NH_4_-N (12.5% TN) present in the effluents. Although the TN concentration was decreased during biological treatment, the decomposition of organic matter introduced NH_4_-N into the liquor. 

Ammonia (NH_3_) and ammonium (NH_4_^+^) are closely related compounds. NH_3_ is a gas that is favored at high pH (>8.0) while NH_4_^+^ is an ion that is favored at low to moderate pH. With air stripping, NH_3_ gas at high pH can easily be volatilized. NH_4_^+^, on the other hand, can rapidly be oxidized to nitrate (NO_3_^−^) aerobically by bacterial action, as shown in the following equation [30,31]:

NH_4_^+^ + 2O_2_ → NO_3_^−^ + H_2_O + 2H^+^, (5)

During biological treatment, air was supplied intermittently. Aerobic conditions favored nitrification and ammonification, while anoxic condition favored denitrification in the reactor. So, there was a conversion of organic N → NH_3_ → NO_3_^−^ → N_2_. Cheng and Liu reported that intermittent aeration (1/1 h) as a post-treatment of anaerobically digested SWW could remove 92% of NH_4_-N and 91% of total Kjeldahl nitrogen (TKN) (Influent: NH_4_-N 221 mg/L, TKN 285 mg/L) [32]. Moreover, The O-P and TP concentrations were reduced in the biological reactor as (141.19 mg/L → 15.44 mg/L) and (172.13 mg/L → 51.23 mg/L), respectively. Most of the initial P in the SWW used in this study was O-P (82%). The majority of the O-P (89.1%) was removed in the biological treatment reactor, while TP removal efficiency was 70.2%. P removal in the biological reactor might be mainly due to the P-accumulating organisms those store P in both aerobic and anoxic conditions. A laboratory experiment conducted by Luo et al. revealed that continuous aeration at low redox potentials using aeration rate of 0.0667 L/min for SWW treatment, removed 32% NH_4_-N, 24% TKN, 26% organic C, and 75% O-P within 24 h, while the removal efficiency was reported to be approximately half in case of intermittent aeration compared to continuous aeration [10]. The high strength SWW was used in this study showed higher removal efficiencies compared to the removal efficiencies reported by Luo et al. [10] and lower than that of Cheng and Liu [32].

The initial sTOC concentration in the influent was 16,796 mg/L. In biological treatment, sTOC removal efficiency was found to be 66.8% with a remaining concentration of 5572 mg/L. sTOC is any compound having C atoms except CO_2_ and associated compounds (CO_3_^2−^, HCO_3_^−^ etc.) [33]. Most of the dissolved organic carbon (DOC) in wastewater are carbohydrates, proteins, fulvic acids, phenols, humic substances, and organic peroxides [34] and serve as the energy source for the microorganisms. Aerobic oxidation of organic C takes place according to the following equations [35].

CH_2_O + O_2_ → H_2_O + CO_2_, (6)

RNH_2_ + H_2_O + H^+^ → ROH + NH_4_^+^, (7)

Initially, 44,216 color units were present in the SWW. After biological treatment, almost half of the color units were removed with the removal efficiency of 46.9% and remaining 23,481 color units were treated in the later stages.

#### 3.1.2. Struvite Production Unit

The NH_4_-N and TN were removed as (989 mg/L; 7891 mg/L) → (549 mg/L; 4357 mg/L) respectively, in the struvite crystallization reactor and the removal efficiencies of NH_4_-N and TN for the struvite crystallization units were almost similar, i.e., 44.5% and 44.8%, respectively. The O-P and TP were removed as (15.4 mg/L; 51.2 mg/L) → (5.9 mg/L; 31.7 mg/L) and were recovered as struvite in the second process with a removal rate of 61.4% O-P and 38.1% TP. In the struvite production unit, struvite was precipitated as dense fine particles. During struvite precipitation, organic N and P might be removed from the SWW and resulted in higher removal of TN and TP than the theoretical value. The sTOC concentrations were decreased to 2725 mg/L from 5572 mg/L with a removal efficiency of 51.1%. The color units from the biological reactor were 23,481; those were decreased, and 12,737 color units remained. The removal efficiency of 45.8% for color units was obtained at the struvite crystallization unit. At this stage, 10,743 color units were removed, which accounted for 24% of the total color units present in the SWW.

**Figure 4:**
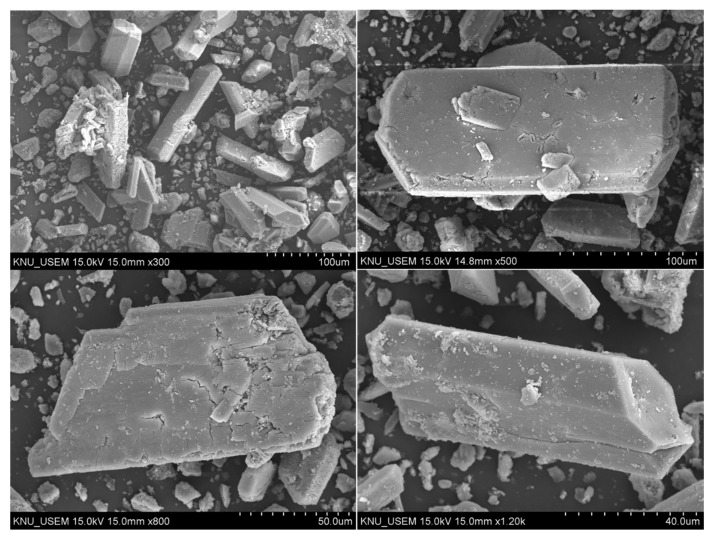
Scanning electron micrograph (SEM) of the recovered material.

**Figure 5:**
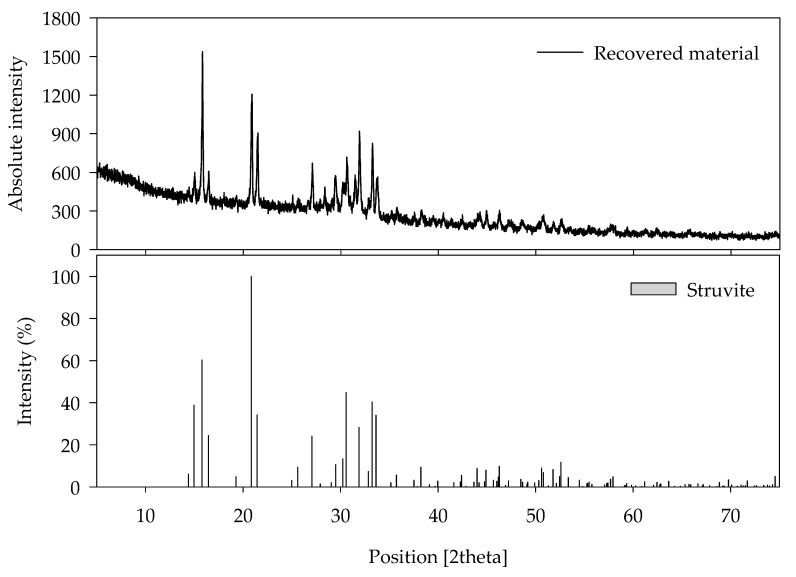
X-ray diffractogram (XRD) comparing recovered material with pure struvite.

Struvite formation takes place when equimolar concentrations of Mg^2+^, NH_4_^+^, and PO_4_^3−^ obtain supersaturated levels. Supersaturation is affected by the pH and ionic constituents of the solution. As the pH of the wastewater increases, the struvite solubility decreases and causes precipitation [36]. The precipitates obtained at the bottom of struvite production unit contained 65.4% organic matter, and 34.6% was inorganic matter in which 23.2% was P. The P_2_O_5_ content of recovered material (53%) was 1.7 times higher than the P imported from China (32%) and Vietnam (27%). Table 3 shows that the concentration of some selected heavy metals in the recovered struvite was under the threshold level suggested by the Korean regulatory authority for fertilizer and feedstock [37]. Apart from using as fertilizer, the recovered struvite could therefore be used in animal feeds as an alternative P source [15,16]. The recovered materials were initially characterized using SEM and then by XRD analysis. The crystals had the morphology of white, rectangular, orthorhombic, and irregular shape in SEM analysis (Figure 4). The XRD pattern of the purified struvite had similarity with reference material that matched with the position and intensity of the peaks (Figure 5).

#### 3.1.3. Electrochemical Reactor

During electrochemical treatment, the pH started declining, and within 16 h, it was 7.8. After 16 h of electrochemical treatment, the TS removal efficiency was found to be 14.0%, whereas the SS removal efficiency was quite high (84.6%) (Table 2), might be due to electroflotation [38]. While a sharp decrease in TP concentration was also observed with increase in reaction time (Table 2). 

**Figure 6:**
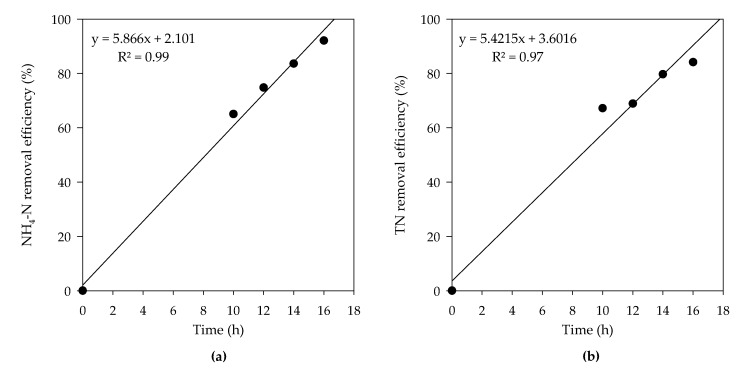
Time lapsed (**a**) NH_4_-N and (**b**) TN removal efficiencies in electrochemical treatment.

Moreover, in electrochemical treatment, not only NH_4_-N but TN was also drastically decreased with the removal efficiencies of 92.1% and 84.1%, respectively after 16 h of reaction time. A linear increase of removal efficiency was seen for the NH_4_-N and TN as reaction time was increased (Figure 6a,b). The removal of N species in the electrochemical reactor was taken place according to following equations:

NO_2_^−^ + HOCl → NO_3_^−^ + Cl^−^ + H_2_O, (8)

2NH_4_^+^ + 3HOCl → N_2_ + 5H^+^ + 3Cl^−^ + 3H_2_O, (9)

**Figure 7:**
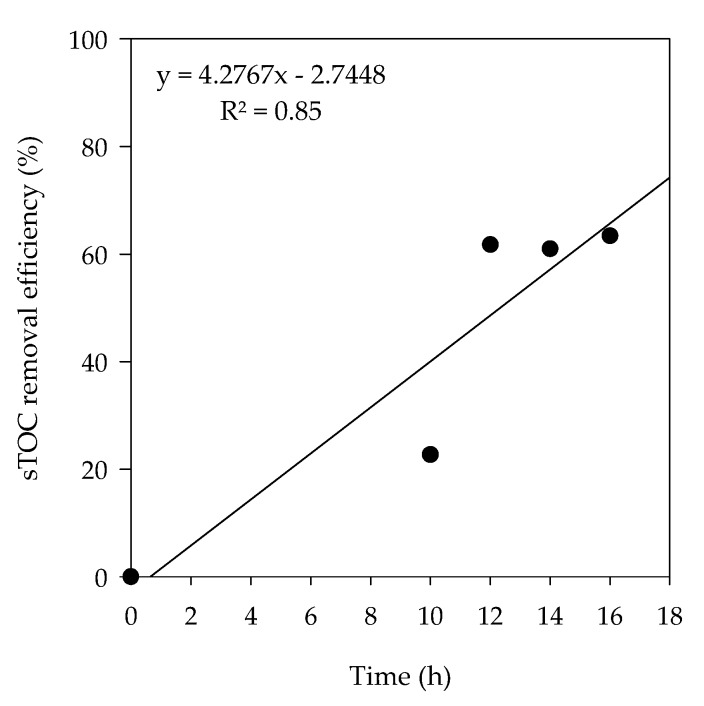
Time lapsed sTOC removal efficiency in electrochemical treatment.

The overall sTOC removal efficiency was found to be 63.4%, with no significant increase in removal efficiency after 12 h (Figure 7). The removal of organic matter by electrochemical decomposition was taken place by the action of hypochlorite ion. Hypochlorite that prevails at the alkaline pH oxidizes the organic matter into carbon dioxide (CO_2_) and inorganic ions [21,39].

OCl^−^ + R → CO_2_ + inorganic ions + H^+^ + e^−^, (10)

OCl^−^ + RCl → R, (11)

**Figure 8:**
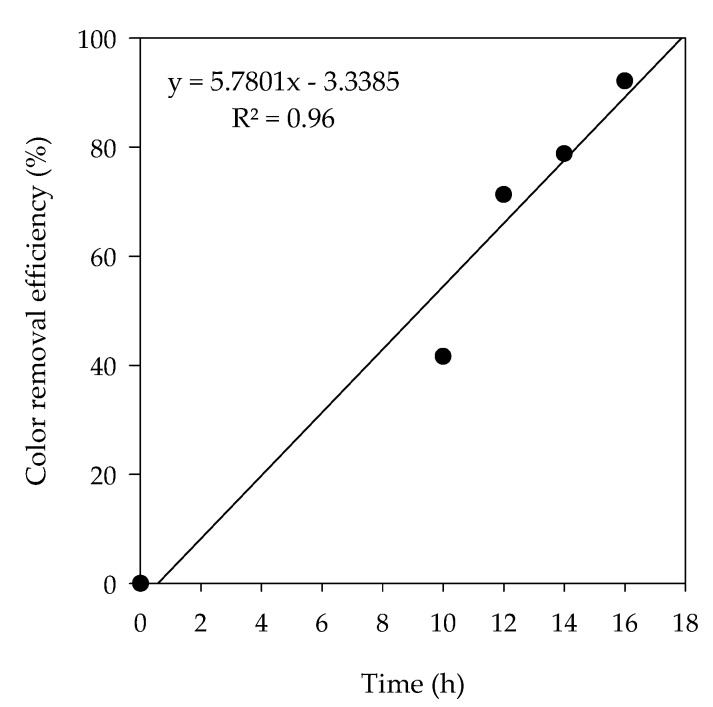
Time lapsed color removal efficiency in electrochemical treatment.

The color was removed by the electrochemical method as electrolysis breaks down the double bonds and benzene ring in pollutants facilitating color removal in wastewater. Color removal was increased as the time of exposure was increased. The removal efficiencies were 41.6%, 71.3%, 78.8%, and 92.1% for 10 h, 12 h, 14 h, and 16 h of reaction time, respectively (Figure 8). Lei and Maekawa reported the removal of 100% NH_4_-N, 51% sTOC, and 95.5% turbidity during electrochemical treatment of anaerobic digestion effluent using Ti/Pt-IrO_2_ electrode at 1 A electric current and 1% NaCl [38]. Cho et al. optimized an electrochemical process for SWW treatment using Ti/IrO_2_ electrode at 7 V electric current and 0.05% NaCl with 6 h HRT and observed removal efficiencies of 99%, 94%, 59%, 64%, and 93% were reported for NH_4_-N, soluble-N, PO_4_-P, sTOC, and, color, respectively[28].

**Figure 9:**
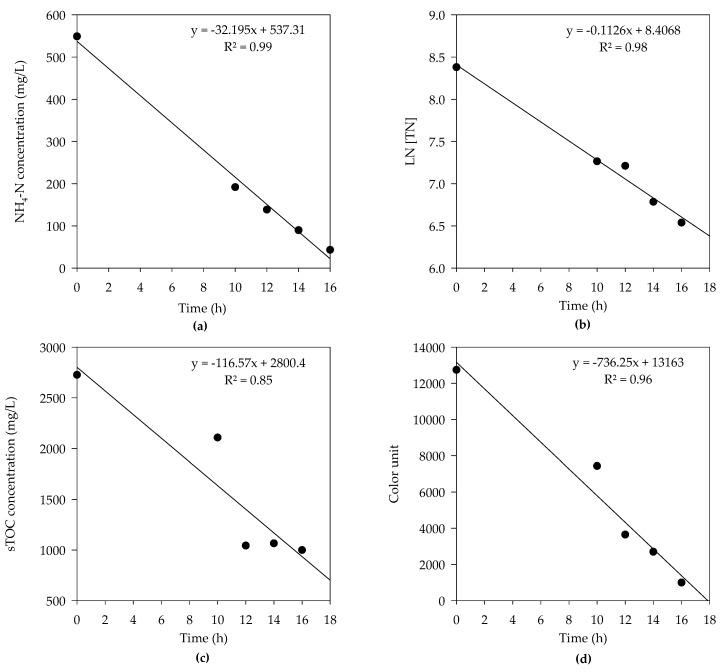
Pseudo zero-order and pseudo first-order kinetics of the electrochemical treatment for (**a**) NH_4_-N, (**b**) TN, (**c**) sTOC, and (**d**) color.

Figure 9a–d shows that the concentrations of NH_4_-N, TN, and color were decreased linearly with time except sTOC. The obtained slopes of the lines were −32.2, −0.1126, −116.6, and −736.2 for NH_4_-N, TN, sTOC, and color, respectively, with respective correlation coefficients of 0.99, 0.98, 0.85, and 0.96 (Figure 9a–d). 

The obtained reaction rates were 32.2 mg-NH_4_-N/L·h, 0.1126 mg-TN/L.h, 116.6 mg-sTOC/L·h, and 736.2 color units/L·h. The NH_4_-N and TN followed pseudo zero-order kinetics and pseudo first-order kinetics, respectively. The NH_4_-N was fitted well (R^2^ = 0.99) on pseudo zero-order kinetics and TN was fitted well (R^2^ = 0.98) on pseudo first-order kinetics, while the sTOC and color removal were also fitted well on pseudo zero-order kinetics.

Pseudo zero-order reaction = −d[C]/dt = k,(12)

Pseudo first-order reaction = −d[C]/dt = k[C] [Cl_2_], (13)

where d[C]/dt is the rate of reaction (mg-reactant/L·h), [C] is the reactant, [Cl_2_] is the chlorine generated during electrochemical process, and k is the reaction constant. 

In this study, the removal of NH_4_-N and sTOC followed pseudo zero-order kinetics, while previous studies reported that the rate of reaction for ammonia and sTOC can follow both pseudo zero-order kinetics and pseudo first-order kinetics as well. Li and Liu stated that Cl^−^ concentrations, current density, and pH, etc. can control the efficiency of N removal from waste streams and observed a pseudo zero-order reaction for ammonia removal in an electrochemical reaction [40], while Priya et al. [41] found contrasting results from this study. They observed that electrochemical treatment of landfill leachate for ammonia and sTOC removal followed a pseudo first-order reaction. Szpyrkowics et al. [42] also reported a pseudo first-order reaction for electrochemical treatment of ammonia in tannery wastewater. 

**Figure 10:**
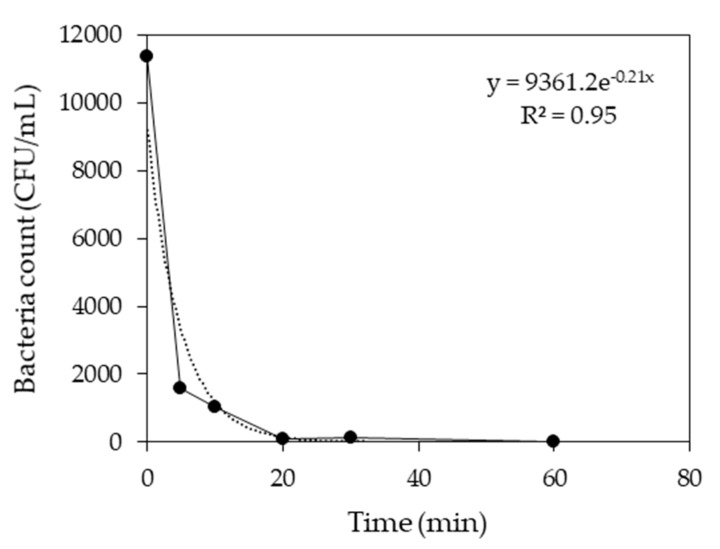
Microbial load analysis of effluent after electrochemical treatment.

For the microbiological analysis, samples were taken at 0, 5, 10, 15, 20, 30, and 60 min intervals from the electrochemical reactor to determine the sterilizing capacity of the electrolysis. As the electrolysis progressed, around 86% bacteria were killed within 5 min and showed a high killing rate of about 99% after 20 min (Figure 10). Matsunaga et al. used C cloth electrodes in an electrochemical reactor that killed 98% of *E. coli* (indicator bacteria) within 10 min in drinking water [43]. Similarly, Okochi et al. [44] conducted an experiment using graphite electrode and found that all the *Vibreo alginolyticus* present in the seawater were died within 10 min. In this study, the removal time was 20 min, almost double of the reported times in other studies. SWW generally contains different types of pollutants and the presence of other pollutants in the SWW might be responsible for the elongated removal time.

### 3.2. Performance of the Farm-Scale Treatment Process

Upscaling of the lab-scale process was done to evaluate the performance of the integrated process under the real farm conditions. The overall removal efficiency of the farm-scale process from SWW to the electrochemical reactor can be seen in Table 4A–D. The SWW produced at the farm was of low strength compared to SWW used in the lab-scale process. The removal of solids was very high as 91.5% of 16.6 g/L TS, and 99.6% of 12.8 g/L SS were removed. Despite being low strength SWW, it had comparatively higher color units (56,056 units) compared to the SWW used for lab-scale experiments. The overall removal efficiencies for NH_4_-N, O-P, TN, TP, sTOC, and color units were 98.6%, 91.9%, 94.5%, 67.0%, 96.1%, and 98.9%, respectively (Table 4). All parameters showed removal rates of over 90% except TP (67%).

The pH of the process was initially decreased in the biological reactor, and then it was increased in the struvite production unit. Finally, the lowest pH was found at the electrolysis stage. Around 82.1–98.5% removal efficiencies were observed in the biological treatment reactor for all parameters except P. The results showed that about 98.5% NH_4_-N, 91% TN, and 90% sTOC were removed during biological treatment. In comparison with the lab-scale test, the O-P and TP removal efficiencies in biological treatment reactor of the farm-scale process were very low. The biological treatment process in the lab was operated with the solid retention time (SRT) and HRT of 20 d along with a settling unit placed after the biological treatment reactor to control mixed liquor suspended solids (MLSS) (Figure 1). In contrast, at the farm-scale, the biological treatment reactor was replaced by SBR with an HRT of 35 d. The long SRT and HRT might lead to oxidation of activated sludge and an increase in P concentration, which ultimately resulted in low O-P and negative TP removal efficiency. Moreover, due to the lack of expertise and ease of operation, the sludge removal unit is not generally installed in the biological treatment process of the swine farms in Korea. Therefore, the increasing sludge volume in the biological treatment tank is controlled by operating at longer SRT to induce endogenous respiration. Although O-P and TP were not removed during biological treatment reactor, 82% O-P and 75% TP was recovered as struvite while TN, sTOCs, and color removal efficiencies were 17%, 34%, and 85% respectively in the struvite production unit. Solids were also removed as 25.6% TS and 67% SS. Further removal of pollutants was done in the electrochemical reactor. The TN, sTOC, and color removal efficiencies were 40%, 42%, and 49% respectively during electrochemical treatment. The effluent generated from this treatment sequence can effectively be discharged into open water bodies. Effluent discharge limits for swine farms were set by Ministry of Environment (MoE), Korea as 120 mg/L, 250 mg/L, and 100 mg/L SS, TN, and TP, respectively [45]. The farm-scale process was found to be under the limit set by the ME (SS, 50 mg/L; TN, 227 mg/L; TP, 22 mg/L).

### 3.3. Overall Performance of the Novel Intregated Treatment Process

SWW contains high SS concentrations that may hinder biological treatment efficiencies. Therefore, separation of solids (mechanical or chemical) prior to biological processes is preferred in the latest treatment techniques [46]. SWW in Korea is often used as soil amendment, and hence, the Korean government has restricted the use of inorganic coagulants in SWW treatment. However, conventional inorganic coagulants are used only if the SWW is discharged into the waterbodies after purification. In this study, solids were removed from the SWW without using mechanical or chemical removal techniques. González-Fernández et al. reported 73% TS removal efficiencies that are lower than this study and comparable SS removal efficiencies using sieving and flocculent (polyacrylamides) [47]. Vanotti et al. also reported 95% SS removal using flocculants [48].

Usually, the swine wastewater contains high levels of N. In the farm-scale study, the overall removal efficiencies for NH_4_-N and TN were 98.6% and 94.5%, respectively. Comparable removal efficiencies were obtained by Ra et al. utilizing sludge separation strategy; those were 97.8% NH_4_-N and 96.7% TKN [49]. Kim et al. also removed 96.2% TN using integrated real-time control strategy [50]. Similarly, Bernet et al. and Won and Ra obtained 94.7% and 100% NH_4_-N removal efficiencies, respectively [51,52]. While Luo et al. found 14–32% NH_4_-N and 24% TN those were quite lower than this study [10]. Lim et al. also reported a 75% TN removal using an ion-exchange biological reactor [1].

The recovery of O-P as struvite was 61% and 82% for lab-scale and farm-scale struvite reactor respectively. Nelson et al. reported an 85% O-P recovery from anaerobic swine lagoon liquid having 50-64 mg/L O-P at pH 9.0 and 1.2:1 Mg:P ratio [53]. Pastor et al. carried out an optimization of a pilot-scale stirred tank reactor that gave O-P precipitations in the range of 40–80% [54]. Several researchers recovered O-P as struvite from SWW with removal efficiencies of 85% [55], 96–98% [56], 63–79% [57], 93% [12,58], and 82% [59] under various optimizing conditions. The struvite recovery in this study was comparable with those reported in the literature.

The removal of sTOC was 94% and 96% in lab-scale and farm-scale processes, respectively. Comparable removal efficiencies of 95.6%, 94.7%, and 90% were reported by Ra et al., Kim et al., and Won and Ra [49,50,52], respectively. Slightly lower sTOC removal efficiencies of 85%, 26.5%, and 88.7% were also reported by Lim et al., Luo et al., and Bernet et al. [1,10,51].

Wastewater has a characteristic color appearance that makes it aesthetically unacceptable. Color of the wastewater is formed due to conjugated double bonds of organic compounds, such as aldehydes, α, β-acivinilalcohols, ketones, dienes, and benzene ring containing several organic pollutants [23]. As microorganisms grow rapidly in the biological reactor, they utilize organic compounds. Hence, the color of wastewater is also decreased with decreased organics. Volatile organic compounds were also evaporated due to air stripping. The color of SWW was completely removed as it passed through the novel integrated sequence and a colorless effluent was obtained at the end. The process removed 97.7–98.9% color units from SWW in lab-scale and farm-scale processes. Won et al. [23] reported a 96.9% color removal using electrochemical treatment from biological secondary treated milking center wastewater. Barrera-Diaz et al. reported the treatment of an industrial effluent having 3750 Pt/Co units color by combined electrocoagulation-γ-irradiation that removed 78% color units [60]. Yetilmezsoy et al. observed an 89% color removal from pretreated poultry wastewater by electrocoagulation using Al electrode at the current density of 25 mA/cm^2^, pH: 8.2, and 1 g NaCl within 20 min [61]. 

## 4. Conclusions

In this study, a novel integrated sequence was proposed for the efficient removal of high strength pollutants from SWW. The intermittent aeration in the biological reactor controlled the oxic and anoxic conditions and removed the TN, TP, and sTOC effectively. NH_4_-N and O-P were precipitated in the struvite production unit as magnesium ammonium phosphate (MgNH_4_PO_4_.6H_2_O) with MgCl_2_ addition and at the end, the electrochemical reactor showed sophisticated removal of pollutants. Intermittent aeration decreased 30.5% TN, 70% TP, 66% sTOC, and 47% color units in the lab-scale test while in the farm-scale process, a removal of 90% TN, 89% sTOC, 86% color units, and no TP removal was obtained. In struvite production unit, the O-P recovery rate of 61.4% and 82% in the form of crystal was achieved in the lab-scale and farm-scale processes, respectively. Recovered struvite showed heavy metal concentrations within the range of the Korean standard for fertilizers and animal feeds. In the farm-scale process, the electrochemical treatment for 10 h was found to be enough to eliminate remaining pollutants, remove color, and disinfect effluent that met the Korean SWW effluent discharge limit. Moreover, the electrochemical removal of NH_4_-N, sTOC, and color followed pseudo zero-order kinetics, while TN removal followed pseudo first-order kinetics. Through the entire treatment process, SWW was converted to liquid compost as a quick-acting fertilizer, struvite as a slow-release fertilizer at the same time, and become safe to dispose into the environment. The novel integrated treatment sequence suggested in this study enables the use of produced liquid compost and struvite as fertilizer during the growing season, while electrochemical treatment allows the safe discharge of SWW in other seasons. Therefore, the proposed sequence of processes can be considered as a solution of SWW management and for simultaneous nutrients (N and P) removal and recycling.

## Figures and Tables

**Table 1:** Characteristics of swine wastewater.

Parameters (mg/L)	Value	
	(A) Lab-Scale	(B) Farm-Scale
TS ^1^ (g/L)	28.52 ± 8.24	16.58 ± 1.66
SS ^2^ (g/L)	11.20 ± 2.02	12.89 ± 2.55
NH_4_-N ^3^	3453.92 ± 67.19	1662.28 ± 181.34
O-P ^4^	141.19 ± 13.64	49.2 ± 23.92
TN ^5^	11362.1 ± 0.64	4139.32 ± 438.84
TP ^6^	172.13 ± 11.03	65.59 ± 13.76
sTOC ^7^	16,769.17 ± 439.12	2096.33 ± 385.04
Color (color units)	44216.39	56,056.57
pH	7.53 ± 0.11	7.53 ± 0.11

^1^ TS, total solids; ^2^ SS, total suspended solids; ^3^ NH_4_-N, ammonium nitrogen; ^4^ O-P, ortho-phosphate; ^5^ TN, total nitrogen; ^6^ TP, total phosphorus; ^7^ sTOC, soluble total organic carbon.

**Table 2:** Characteristics of effluent according to unit processes in the lab-scale.

Parameters (mg/L)	Influent	Biological Treatment	Struvite Production Unit	Electrochemical Treatment				Removal (%)				
	(A)	(B)	(C)	(D)	(E)	(F)	(G)	(A-B)	(B-C)	(C-E)	(A-E)	(A-G)
				10 h	12 h	14 h	16 h					
TS ^1^ (g/L)	28.52	13.52	6.84	7.42	6.38	6.28	5.88	52.7	49.4	6.7	77.6	79.4
	±8.24	±0.54	±0.31									
SS ^2^ (g/L)	11.20	2.51	0.91	0.5	0.26	0.52	0.14	77.6	63.7	71.4	97.7	98.8
	±2.02	±0.57	±0.07									
NH_4_-N ^3^	3453.92	988.96	548.84	191.87	138.37	89.99	43.33	71.4	44.5	74.8	96.0	98.7
	±67.19	±96.95	±49.36	±0.16	±0.47	±0.14	±0.02					
O-P ^4^	141.19	15.44	5.96	6.54	6.57	6.12	7.48	89.1	61.4	-	95.3	94.7
	±13.64	±2.93	±0.59	±0.07	±0.07	±0.07	±0.14					
TN ^5^	11362.1	7891.34	4357.32	1429.34	1355.14	884.8	691.28	30.5	44.8	68.9	88.1	93.9
	±0.64	±0.59	±0.06									
TP ^6^	172.13	51.23	31.72	21.51	14.07	13.9	11.98	70.2	38.1	55.6	91.8	93.0
	±11.03	±2.31	±0.17									
sTOC ^7^	16,769.17	5571.93	2725.61	2107.65	1043.1	1065.4	998.93	66.8	51.1	61.7	93.8	94.0
	±439.12	±648.10	±378.50	±4.03	±77.22	±38.32	±49.50					
Color (color units)	44216.39	23481.28	12737.64	7434.85	3654.57	2700.6	1001.79	46.9	45.8	71.3	91.7	97.7
pH	7.53	8.68	8.66	8.27	8.53	8.02	7.81	-	-	-	-	-
	±0.11	±0.10	±0.07									

^1^ TS, total solids; ^2^ SS, total suspended solids; ^3^ NH_4_-N, ammonium nitrogen; ^4^ O-P, ortho-phosphate; ^5^ TN, total nitrogen; ^6^ TP, total phosphorus; ^7^ sTOC, soluble total organic carbon.

**Table 3:** Concentration of selected heavy metals in the recovered material and the threshold limits of heavy metals in fertilizer and feedstock suggested by the Korean Regulatory Authority.

Parameters	Recovered Material (mg/kg)	Standard Limits (mg/kg)	
		Fertilizer	Feedstock
As	0	45	2
Cd	0	5	1
Cr	0	200	100
Cu	17.86	360	−
Pb	0	130	10
Hg	ND ^1^	2	0.4
Ni	0	45	−
Se	ND ^1^	−	2
Zn	95.54	900	−

^1^ ND = not detected.

**Table 4:** Characteristics of effluent according to unit processes in the farm-scale.

Parameters (mg/L)	Influent	Biological Treatment	Struvite Production Unit	Electrochemical Treatment	Removal (%)			
	(A)	(B)	(C)	(D)	(A-B)	(B-C)	(C-D)	(A-D)
				10 h				
TS ^1^ (g/L)	16.58	2.97	2.21	1.41	82.1	25.6	36.2	91.5
	±1.66	±0.04	±0.04	±0.03				
SS ^2^ (g/L)	12.89	1.58	0.52	0.05	87.7	67.1	90.4	99.6
	±2.55	±0.07	±0.02	±0.00				
NH_4_-N ^3^	1662.28	24.7	25.12	23.06	98.5	-	6.5	98.6
	±181.34	±10.69	±7.57	±14.49				
O-P ^4^	49.2	43.83	7.9	3.99	10.9	82.0	49.5	91.9
	±23.92	±12.78	±3.71	±1.47				
TN ^5^	4139.32	375.86	310.43	226.97	90.9	17.3	39.6	94.5
	±438.84	±94.33	±88.47	±30.65				
TP ^6^	65.59	72.2	17.89	21.65	−10.1	75.2	-	67
	±13.76	±9.09	±5.49	±4.46				
sTOC ^7^	2096.33	212.78	140.98	82.46	89.8	33.7	41.8	96.1
	±385.04	±57.30	±42.43	±25.24				
Color (color units)	56,056.57	7811.57	1180.34	603.59	86.1	84.9	48.9	98.9
pH	7.53	7.31	7.56	7.24	-	-	-	-
	±0.11	±0.10	±0.07					

^1^ TS, total solids; ^2^ SS, total suspended solids; ^3^ NH_4_-N, ammonium nitrogen; ^4^ O-P, ortho-phosphate; ^5^ TN, total nitrogen; ^6^ TP, total phosphorus; ^7^ sTOC, soluble total organic carbon.
